# Dehydration-Driven Changes in Solid Polymer Electrolytes: Implications for Titanium Anodizing Efficiency

**DOI:** 10.3390/ma18153645

**Published:** 2025-08-03

**Authors:** Andrea Valencia-Cadena, Maria Belén García-Blanco, Pablo Santamaría, Joan Josep Roa

**Affiliations:** 1 Steros GPA Innovative S.L., C/Maracaibo 1, 08030 Barcelona, Spain; avalenca32@alumnes.ub.edu; 2 Department of Materials Science and Physical Chemistry, Facultat de Química, Universitat de Barcelona, C/Martí i Franquès 1, 08028 Barcelona, Spain; 3 CIDETEC, Basque Research and Technology Alliance (BRTA), P. Miramón, 196, 20014 Donostia-San Sebastián, Spain; bgarcia@cidetec.es

**Keywords:** solid polymer electrolyte, thermal aging, dehydration, dry anodizing, titanium anodizing

## Abstract

This study investigates the thermal stability and microstructural evolution of the solid electrolyte medium used in DLyte^®^ dry electropolishing and dry anodizing processes. Samples were thermally aged between 30 °C and 45 °C to simulate Joule heating during industrial operation. Visual and SEM analyses revealed shape deformation and microcrack formation at temperatures above 40 °C, potentially reducing particle packing efficiency and electrolyte performance. Particle size distribution shifted from bimodal to trimodal upon aging, with an overall size reduction of up to 39.5% due to dehydration effects, impacting ionic transport properties. Weight-loss measurements indicated a diffusion-limited dehydration mechanism, stabilizing at 15–16% mass loss. Fourier transform infrared analysis confirmed water removal while maintaining the essential sulfonic acid groups responsible for ionic conductivity. In dry anodizing tests on titanium, aged electrolytes enhanced process efficiency, producing TiO_2_ films with improved optical properties—color and brightness—while preserving thickness and uniformity (~70 nm). The results highlight the need to carefully control thermal exposure to maintain electrolyte integrity and ensure consistent process performance.

## 1. Introduction

Titanium and its alloys, particularly Grade 5 (Ti-6Al-4V), have emerged as critical materials in a wide range of high-performance applications due to their unique combination of mechanical and physicochemical properties [1]. This alpha-beta titanium alloy is widely recognized for its exceptional strength-to-weight ratio [2], superior corrosion resistance [3], high fatigue resistance, and excellent biocompatibility [4,5]. These characteristics make it indispensable in demanding sectors such as aerospace, where weight reduction without compromising strength is essential [6]; in medical implants requiring both mechanical integrity and long-term biocompatibility [4,7]; and in the automotive industry, where corrosion resistance and structural performance are vital for advanced components [8].

To further optimize these surface-related properties, anodizing is frequently applied to titanium substrates. Anodizing is an electrochemical treatment that modifies the metal’s surface by forming a controlled titanium dioxide (TiO_2_) layer [9]. This oxide layer significantly enhances the material’s corrosion resistance and wear performance and enables the generation of various interference colors useful for both aesthetic and functional purposes, such as batch traceability or implant surface recognition [3,10]. The anodizing process traditionally involves immersing titanium workpieces in a liquid electrolyte under applied voltage, promoting oxide growth via field-assisted oxidation mechanisms. The resulting oxide layer’s thickness, uniformity, adhesion, and morphology depend on several interrelated parameters: applied voltage, current density, electrolyte composition, processing time, and—especially—temperature [9].

Temperature plays a particularly decisive role in conventional wet anodizing processes, directly influencing the kinetics of electrochemical reactions and the subsequent oxide growth dynamics. Typically, these processes are conducted at temperatures below 30 °C to promote dense, adherent, and uniform oxide layers with low porosity and predictable coloring effects [3,9]. Higher temperatures tend to accelerate reaction kinetics excessively, leading to oxide porosity, decreased film adhesion, cracking, and instability in the interference colors produced—undesirable defects in critical applications such as implants or aerospace fasteners [3]. Grade 5 titanium poses additional challenges, as its alloying elements (6% aluminum and 4% vanadium) alter oxide growth rates, stress development, and crystallinity, further complicating the control of the anodizing process and its temperature sensitivity [4,5,9].

In this context, the DryLyte^®^ Technology offers a breakthrough alternative to conventional anodizing by eliminating liquid electrolytes entirely and instead employing a proprietary dry-anodizing medium composed of solid-state electrolyte granules [11]. In this context, both the structural characteristics and the nature and degree of functionalization of the resin play a crucial role in its ion-exchange behavior. Morphologically, ion-exchange resins are typically classified into two main types: microporous (or macroreticular) resins and gel-type resins.

- Macroporous resins possess a stable, porous structure even in the dry state. Their synthesis generally requires a minimum cross-linking degree of approximately 10%. These resins appear opaque due to their complex, multi-channeled bead architecture, which provides a highly effective surface area. However, as reported in [12], they are more mechanically fragile compared to gel-type resins.
- Gel-type resins consist of a three-dimensional (3D) polymer network swollen with a solvent that is uniformly distributed throughout the structure. Upon drying, the gel contracts, and its porosity becomes negligible. These resins typically exhibit heterogeneous micropority, with pore sizes ranging from 0.7 to 2 nm, and a cross-linking degree between 4 and 10%. Visually, they are characterized by a translucent appearance [13].

This innovative system enables anodizing reactions under dry conditions, where ionic transport and oxide growth are governed by a controlled solid electrolyte matrix rather than temperature-sensitive aqueous solutions. As a result, the DryLyte process is fundamentally less dependent on temperature fluctuations, offering greater consistency in oxide layer characteristics, including thickness, adhesion, and interference in color development [11].

One of the DryLyte^®^ technology’s most significant advantages over traditional methods is its reduced thermal sensitivity, which simplifies process control and eliminates the need for precise thermal regulation systems common in wet anodizing setups [11]. Unlike liquid-based systems that require complex chilling equipment and tight temperature monitoring to ensure film quality and color reproducibility, the solid-state mechanism of DryLyte^®^ permits stable and reliable oxide layer formation across a wider temperature window [11]. This robustness not only reduces operational costs and energy consumption but also opens opportunities for deploying the technology in industrial-scale or field environments where tight temperature control is impractical [11].

Despite these benefits, one critical issue that remains underexplored is the long-term durability of the solid electrolyte medium itself. Prolonged anodizing operations or exposure to thermal cycling, mechanical loading, and electrochemical stress may lead to gradual degradation of the solid electrolyte matrix—affecting its ionic conductivity, homogeneity, and ultimately its effectiveness in producing high-quality oxide layers [11]. Understanding the degradation mechanisms of this solid electrolyte is essential for predicting the system’s service life, optimizing process parameters, and ensuring sustained performance in demanding industrial applications such as aerospace component finishing or biomedical implant anodization [4,7,11].

The present research aims to systematically investigate the influence of temperature on the DryLyte^®^ anodizing process, with a particular focus on the behavior and degradation of the solid electrolyte under varying thermal conditions. By conducting a series of controlled anodizing trials at different temperatures and thoroughly analyzing the resulting oxide layer properties—morphology, thickness, composition, and adhesion—this study seeks to define the process window that maximizes electrolyte longevity and oxide film quality. Additionally, the mechanisms of electrolyte wear, ion depletion, and structural alteration will be examined to propose strategies for mitigating degradation effects, thereby improving process reliability and sustainability for long-term industrial deployment [11].

## 2. Materials and Methods

### 2.1. Materials

The base material was Ti6Al4V (titanium alloy) Grade 5 (hereafter referenced to as Ti5 or the alloy), supplied by KLEIN S.R.L (Milan, Italy) in the form of cylinders 10 mm in diameter and 20 mm in length, with an M6 thread hole on one base for electrical contact.

The anodizing process was performed using a commercial high-concentration sulfuric acid electrolyte (8 wt.% H_2_SO_4_, hereafter denoted as H-H_2_SO_4_) to ensure consistent electrochemical conditions, as described in [11]. This concentration was selected to: (1) maximize ionic conductivity while minimizing excessive acid-induced dissolution of the oxide layer; (2) evaluate Joule heating effects under high current densities, where resistive heating (I^2^·R, with R being the resistivity of the solid electrolyte media) induces measurable temperature gradients in the solid electrolytes, impacting thermal stability. The exothermic nature of anodization in concentrated H_2_SO_4_ intensifies temperature fluctuations, potentially influencing pore uniformity and oxide growth kinetics.

### 2.2. Dry-Anodizing Process

**Figure 1:**
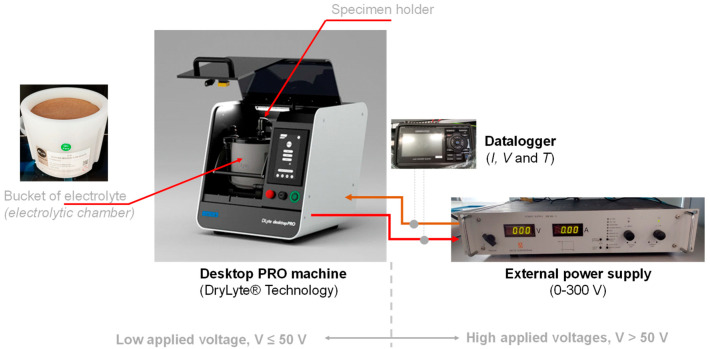
Schematic representation of the experimental setup used for sequential dry electropolishing and anodizing. In the diagram, I, V, and T represent current intensity, voltage, and temperature, respectively. Adapted from [11].

Prior to anodizing, Ti5 cylinders were ultrasonically degreased in pure acetone (Sigma-Aldrich) for 10 min at room temperature and subsequently dried with purified air. To eliminate surface defects from prior processing, dry electropolishing was performed using the DryLyte^®^ Technology (DLyte Desktop PRO machine, Barcelona, Spain, see Figure 1) with a rectangular symmetric alternating current (AC) pulse train. The process consisted of three steps, each applying a maximum voltage ranging from 15 to 22 V with a 10 μs positive pulse time (T^+^), followed by a variable negative pulse time (T^−^) between 30 and 100 μs depending on the step. The pause time (T_p_) between pulses was held constant at 10 μs. The total dry electropolishing time was approximately 60 min.

For anodizing, processes of 2 min at 48V were applied to study the effect of the electrolyte operating time (S1–S7); and for corrosion analysis, the processes were performed at 20 V and 30 V for 2 min.

As shown in Figure 1, in situ temperature measurement was performed using a Pt100 probe (Farnell Components S.L., Barcelona, Spain) to correlate Joule heating with optimized anodizing parameters as established in a preliminary study [11].

### 2.3. Characterization Techniques

#### 2.3.1. Color Characterization

The optical properties of the anodized TiO_2_ layers were quantitatively evaluated using a CM-2600d spectrophotometer (Konica Minolta, Japan) with a D65 standard illuminant. Color measurements as a function of temperature were carried out in the CIELAB (Lab*) color space, an internationally standardized colorimetric system, where: (1) L* represents luminance (0 = pure black, 100 = diffuse white); (2) a* denotes the green (*−a*) to magenta (*+a*) chromaticity axis, and (3) b* indicates the blue (*−b*) to yellow (*+b*) chromaticity axis [14].

All measurements were performed at fixed 10° illumination and viewing angles to ensure consistency. Complementary qualitative color analysis was performed using optical microscopy (Nikon Epiphot200, Berlin, Germany) to verify the macroscopic appearance of the dry anodized specimens as a function of the testing temperature.

#### 2.3.2. Chemical Analysis of the Electrolyte

The chemical composition and structural integrity of the polymeric ion-exchange electrolytes at various testing temperatures were characterized using Fourier transform infrared spectroscopy (FTIR, Jasco Analytical Instruments, Japan), simulating service-like conditions and revealing information about functional groups. Measurements were conducted using a high-precision Jasco FT/IR-6000 series spectrophotometer (Jasco Analytical Instruments, Japan) equipped with a cesium iodide (CsI) beam splitter for mid-infrared analysis.

The spectrometer was configured to acquire spectra across the fundamental molecular vibration region (4000–500 cm^−1^) with a resolution of 4 cm^−1^, achieved through careful optimization of the interferometer mirror velocity and aperture settings. Each measurement consisted of 128 accumulated scans to ensure an adequate signal-to-noise ratio while maintaining spectral fidelity. Prior to sample analysis, background scans were collected under identical conditions using a dry-air-purged sample chamber to compensate for atmospheric CO_2_ and water vapor interference.

The resulting spectral data were processed using Spectra Manager^TM^ software 2.15.01 (Jasco Scientific) with atmospheric compensation and baseline correction algorithms to ensure accurate peak assignment and quantitative comparison between samples.

#### 2.3.3. Microstructural Characterization

The thickness of the anodic TiO_2_ layer was determined using an indirect reflectometric approach performed with a Filmetrics F-20 thin-film measurement system (KLA Corporation, San Diego, CA, USA). This instrument uses a broadband ultraviolet (UV)–visible light source coupled with high-resolution spectrometer to detect interference patterns in the reflected light spectrum. Layer thickness was calculated from the spectral fringe positions using proprietary modeling software, considering the optical constants of TiO_2_.

To validate the reflectometric measurements, direct thickness assessments were conducted via cross-sectional analysis using a dual-beam focused ion beam/field emission scanning electron microscope (FIB/FESEM, Carl Zeiss Neon 40, Zeiss Group, Oberkochen, Germany). This high-resolution technique not only provided accurate thickness verification but also enabled detailed morphological characterization of the anodic oxide layers.

FIB milling was carefully optimized to minimize beam-induced artifacts. Regions of interest were protected with a conformal platinum (Pt) layer via ion-beam-assisted deposition to prevent surface damage. Cross-sections were creating using a gallium (Ga^+^) ion source operated at 30 kV, following a progressive current reduction strategy—from coarse milling at several nA and finishing with a fine polishing step at 500 pA—to yield high-quality, artifact-free surfaces.

#### 2.3.4. Corrosion-Resistance Analysis

Electrochemical measurements were performed at 37 °C in Hank’s solution as electrolyte to simulate in vivo conditions and using a BioLogic VMP3 potentiostat (CL Houten, The Netherlands) with a 3 electrode system. The anodized titanium sample was set as the working electrode, an Ag/AgCl reference electrode and a Pt sheet as the counter electrode. The open circuit potential (OCP) was measured over 1 h. Afterwards, potentiodynamic polarization measurements were performed from −250 mV to 4000 mV (vs. OCP) with a scan rate of 0.167 mV/s.

## 3. Results and Discussion

### 3.1. Electrolyte Evolution as a Function of the Testing Temperature

#### 3.1.1. Visual Inspection

**Figure 2:**
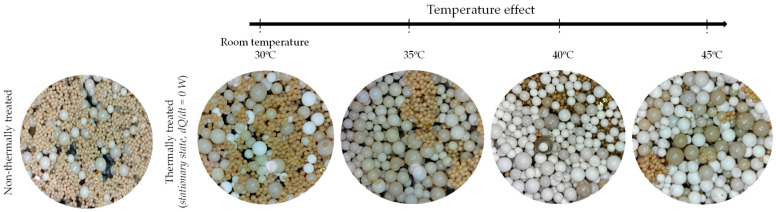
Stereographic microscope images show the evolution of the solid particle as a function of aging temperature, ranging from ambient temperature (left-side image) up to 45 °C.

To systematically investigate the influence of Joule heating effects during electrochemical processing using DLyte^®^ Technology, the solid polymer electrolyte media were subjected to a controlled thermal aging protocol within an environmental chamber equipped with precise temperature and atmospheric regulation systems. The aging temperatures were systematically varied from 30 to 45 °C, in 5 °C increments, to simulate thermal conditions encountered during real-world dry electrochemical and dry anodizing operations, as shown in Figure 2.

This figure provides a detailed stereographic evaluation of the morphological transformation occurring within the solid polymer electrolyte matrix utilized in both dry electropolishing and dry anodizing process, assessed as a function of the applied testing temperature. The solid electrolyte particles were subjected to controlled thermal conditions by placing them inside a laboratory oven with an inert atmosphere, effectively replicating the Joule heating effect typically experienced during operation. This thermal simulation was specifically designed to expose the particles to thermal stress conditions analogous to those encountered during actual service, thereby ensuring the relevance of the observed microstructural changes.

**Figure 3:**
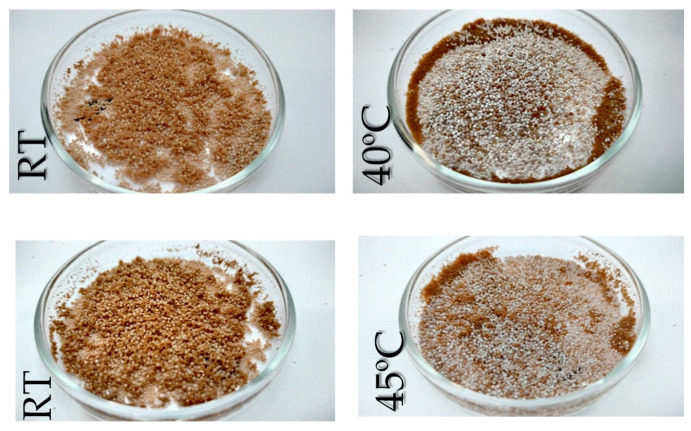
Comparison of untreated (**left**) and heat-treated (**right**) electrolyte at 40 and 45 °C. Upper and bottom are stereographic micrographs.

As shown in Figure 2, when the working temperature exceeds 40 °C, there is a noticeable increase in the number of particles exhibiting a white-toned appearance. Although the material composition remains the same, this color change from brown to white is primarily attributed to temperature-induced chemical and structural modifications occurring both on the surface and within the resin particles. These changes may involve dehydration, or degradation of functional groups, which alter the optical properties of the material. The underlying mechanisms and implications of this transformation will be further discussed in detail in Section 3.1.3. Under these intermediate thermal conditions, the system appears to reach a steady-state behavior, wherein the dehydration-related mechanisms affecting the particles stabilize and no longer progress significantly with further temperature increase. This is clearly illustrated in Figure 3, where samples exposed to 40 and 45 °C exhibit virtually no change in the population of white-toned particles, suggesting a thermal saturation threshold has been reached.

**Figure 4:**
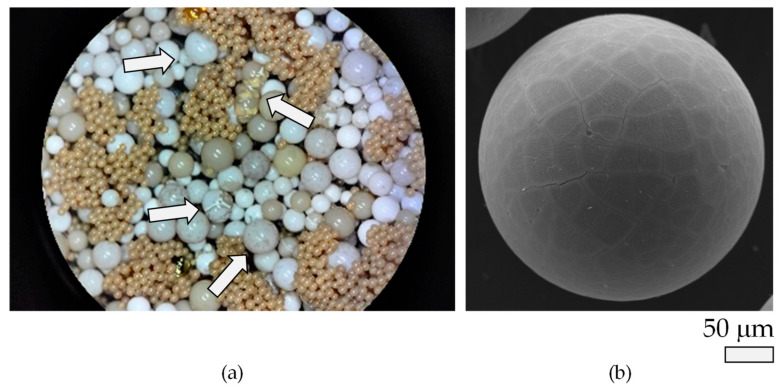
(**a**) Stereographic image of the electrolyte treated at 45 °C upon reaching steady-state conditions (also labelled as the stationary state in Figure 2). The white arrows in Figure 4a indicate deformed particles due to exposure at 45 °C, and (**b**) SEM micrograph of a heat-treated particle following high-temperature exposure (45 °C) and subsequent anodization.

Upon closer inspection using higher magnification techniques, including stereographic microscopy and SEM—as shown in Figure 4a,b, respectively—two distinct thermally induced degradation phenomena become evident.

- At operational temperatures of 40 °C or higher, the particles begin to lose their initial spherical geometry, as indicated by the arrows present in Figure 4a. This deformation negatively impacts the efficiency of both the electropolishing and anodizing process by altering particle packing and surface contact dynamics (see Section 3.1.2).
- Additionally, due to cyclic thermal loads during repeated electropolishing and anodizing treatments, the polymeric matrix undergoes thermal fatigue, resulting in the formation of surface-level microcracks (Figure 4b).

These thermally induced degradation mechanisms lead to significant consequences in both the microstructural evolution (e.g., particle size modification) and the chemical composition of the solid active electrolyte media. The corresponding changes in morphology and material chemistry will be discussed in detail in Section 3.1.2 and Section 3.1.3, respectively.

#### 3.1.2. Particle Size Distribution

A comprehensive statistical deconvolution of the extensive particle size dataset (around 1200 per condition)—measured in terms of particle diameter—enables a robust and quantitative assessment of the particle size distribution as a function of thermal exposure. This analysis focused on comparing two distinct thermal conditions: (1) room temperature, corresponding to as-received (virgin) solid active particles used as a baseline reference, and (2) a test temperature of 45 °C, chosen to simulate service-like operating conditions typically encountered during the dry electrochemical polishing process or dry anodizing process, as highlighted in [11].

**Figure 5:**
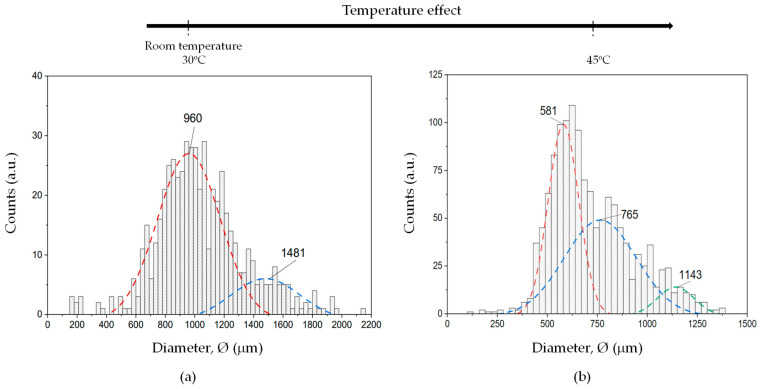
Histogram of the particle size distribution based on diameter measurements, constructed using a uniform bin size of 25 μm, derived from a dataset of 1200 particles for (**a**) untreated and (**b**) particles thermally aged at 45 °C. The simulated particle size distribution function, generated using statistical fitting parameters (summarized in Table 1), is superimposed on the histogram to illustrate the quality of the fit and the underlying distribution model.

The resulting particle size distributions are graphically presented in Figure 5a,b, which display the histograms for the untreated and thermally aged particles, respectively; after treating the raw data using the Ulm and Constantinides method [15,16,17].

Both histograms were constructed using a uniform bin size of approximately 25 μm to ensure consistent resolution across the dataset. These histograms reveal a multimodal distribution profile, indicating the coexistence of multiple particle populations, each centered at different mean diameter values, which are summarized in Table 1.

Notably, the thermal treatment at 45 °C shifts the modal peaks of the distribution, accompanied by a measurable reduction in particle size across both the coarse and fine particle populations. This phenomenon is primarily attributed to dehydration mechanisms occurring within the polymeric matrix of the solid electrolyte particles at intermediate temperatures. As the particles lose physically or chemically bound water (O-H), a slight contraction in their overall dimensions occurs, resulting in a diameter reduction estimated at approximately 39.5 and 22.8% for the fine and coarse fractions, respectively.

This thermally induced shrinkage not only alters the size distribution but may also affect the functional properties of the solid electrolyte, such as packing density, surface contact efficiency, and ionic transport characteristics during electrochemical processing.

As shown in Figure 5a, the particle size distribution for the untreated solid electrolyte media exhibits a distinct bimodal profile, indicating the presence of two predominant particle populations. The fine particle fraction is distributed within a size range of approximately 400 to 1500 μm, whereas the coarse particle population spans from 1000 to 1900 μm. This overlapping range suggests a degree of size dispersion and agglomeration, which may result from the inherent variability in the particle synthesis or processing method used during the fabrication of the raw material.

In contrast, the histogram and the simulated particle size distribution function corresponding to the thermally aged particles subjected to 45 °C (see Figure 5b) displays a significantly more complex distribution profile, characterized by the emergence of three distinct modal peaks, indicative of multimodal behavior. The fine particle population in this case is primarily distributed between 375 and 800 μm, reflecting a contraction in size relative to the untreated counterpart. A newly developed intermediate (medium) particle population, absent in the as-received material, emerges within the range of 250 to 1250 μm, suggesting particle fragmentation or redistribution effects induced by thermal stress. Finally, the coarse particle fraction in the thermally aged sample appears slightly narrowed, with size values ranging from 900 to 1350 μm, potentially because of thermally induced densification or partial structural collapse of the outer shell of the spherical particles, triggered by thermal fatigue mechanisms occurring under service-like working conditions.

The evolution from bimodal to trimodal particle size distribution upon thermal exposure strongly suggests that elevated temperatures induce significant microstructural reorganization in the solid electrolyte media. This includes potential mechanisms such as thermally driven dehydration, surface erosion, polymer chain rearrangement, or even partial sintering at localized contact points between the particles. The broader spread and redistribution of particle size after aging highlights the importance of thermal stability in maintaining a consistent and functional particle morphology, which is critical for ensuring uniformity and reproducibility in electrochemical performance.

#### 3.1.3. Weight Loss Evolution

To conduct a systematic investigation of Joule heating effects during dry electropolishing and dry anodizing processing using the DLyte^®^ Technology, controlled thermal aging experiments were carried out on 16 L of solid polymer electrolyte media. These tests were performed inside a precision-regulated environmental chamber, where both temperature and atmosphere were tightly controlled to simulate real-world, service-like working conditions encountered during dry electropolishing and dry anodizing operations.

The temperature range selected for the study varied incrementally from 30 to 45 °C, using 5 °C increments, corresponding to the expected thermal window under typical industrial electrochemical processing conditions using the DryLyte^®^ Technology [11].

**Figure 6:**
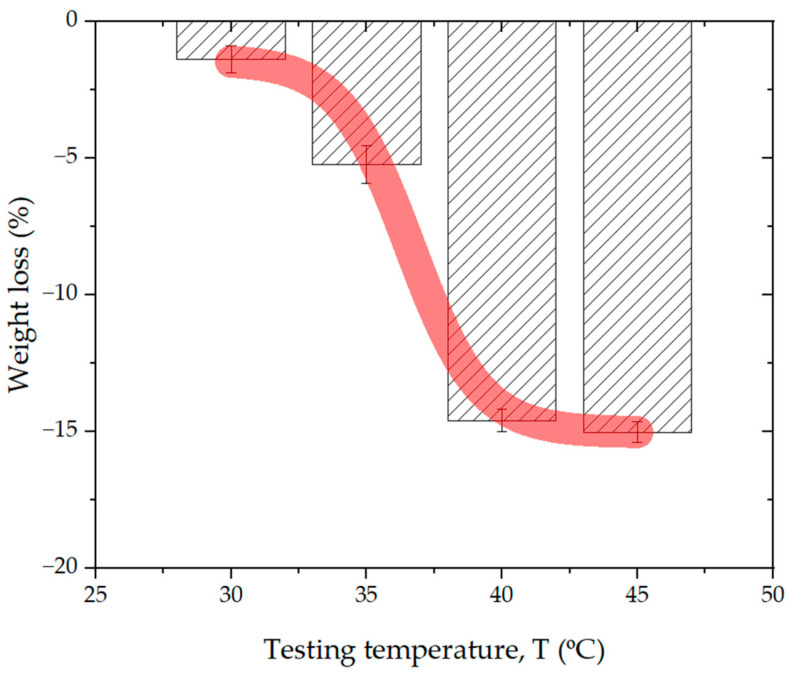
Evolution of weight loss associated with the dehydration of the solid electrolyte media as a function of testing temperature under controlled atmospheric conditions. The fitting curve is overlaid on the experimental data points, shown in semi-transparent red. Data reflect the progressive removal of physically and chemically bound water (O-H) from the solid electrolyte particles during thermal exposure.

The primary focus of this thermal-aging protocol was to investigate the kinetics of dehydration in solid active particles—specifically, the quantification of water loss as a function of temperature. The results of this investigation are illustrated in Figure 6, which shows the evolution of water content in the solid electrolyte media as thermal exposure increases. The dehydration curve exhibits a characteristic sigmoidal behavior (see Equation (1)), typical of diffusion-limited processes, where the rate of water loss accelerates at intermediate temperatures before reaching a plateau. At temperatures near 40 °C, the dehydration process approaches a steady state, with the total water loss stabilizing at approximately 15–16% of the initial water content.

This plateau indicates that the polymeric matrix of the solid electrolyte undergoes a transition to a more thermally stable configuration, where further water loss is kinetically limited or physically constrained by the structure of the matrix. The observed stabilization in water content aligns closely with the morphological changes identified in Figure 2 and Figure 3, where particle coloration and structural features become essentially invariant at or above 40 °C, suggesting the onset of a thermally equilibrated state.

(1) $$Weight\;loss=A_{1}+\frac{\left(A_{2}-A_{1}\right)}{\left(1+10^{(\log\left(X_{0})-Testing\;temperature\right)\cdot p}\right)}$$

where *A*_1_, *A_2_*, log *X_0_* and *p* are fitting parameters, being −15.03; −1.37; 36.59018 and −0.33333, respectively.

Furthermore, this result supports the hypothesis that Joule heating during service operation not only leads to localized thermal fatigue but also significantly alters the state of hydration and mechanical integrity of the particles (see Figure 4b). These findings have critical implications for the long-term performance and reliability of the DLyte^®^ process, particularly in terms of maintaining electrolyte activity, mechanical resilience, and uniform polishing efficiency under extended thermal cycling.

### 3.2. Temperature Effect of the Anodizing Process

**Figure 7:**
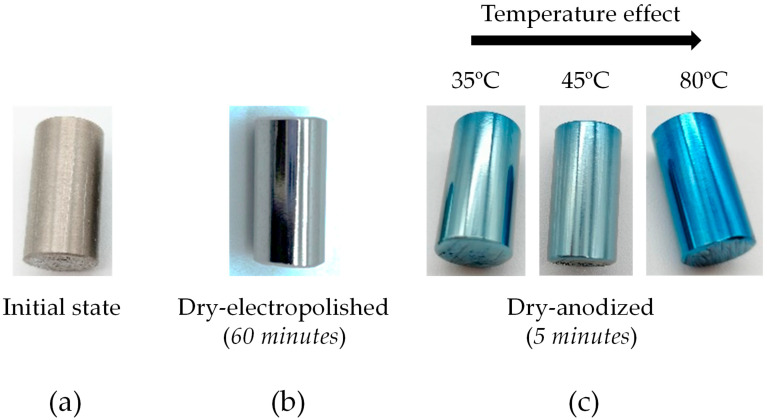
Visual representations of the specimen at various stages: (**a**) initial state; (**b**) after 60 min of dry electropolishing; and (**c**) following dry anodization at a constant applied voltage of 48 V, using solid active electrolyte pre-treated at temperatures ranging from 35 to 80 °C (from left to right).

As shown in Section 3.1, solid active particles undergo dehydration due to the Joule effect, leading to an increased acid concentration within them. Figure 7a,b depict the initial state of a cylinder and its appearance after 60 min of dry electropolishing. Subsequently, a dry anodizing process was conducted to generate a protective titanium oxide (TiO_2_) layer. Notably, the sample treated with electrolyte dehydrated at 80 °C exhibits enhanced color and brightness. This improvement is primarily attributed to the reduced water content in the solid medium responsible for anodization, resulting in a higher acid concentration within the particles. Consequently, a greater current density can traverse the solid particles, interacting more effectively with the material undergoing anodization. These findings align with those reported by Valencia-Cadena et al. [11].

Electrochemically, the dehydration of solid particles reduces their water content, thereby increasing the acid concentration within them. This elevated acid concentration enhances the conductivity of the medium, facilitating a more efficient anodic oxidation process. The increased current density resulting from improved conductivity allows for a more uniform and controlled growth of the TiO_2_ protective layer. Such conditions are conducive to achieving coatings with superior optical properties, as evidenced by the enhanced color and brightness observed in the treated sample. These observations are consistent with the electrochemical principles governing anodization processes as also reported in [11].

Furthermore, the increased acid concentration within the dehydrated particles leads to a higher availability of protons, which can influence the kinetics of the anodization reaction. This can result in a more rapid formation of the oxide layer, contributing to the observed improvements in the coating’s properties. Additionally, the elevated acid concentration may affect the morphology and crystallinity of the TiO_2_ layer, potentially leading to the formation of more uniform and well-defined crystalline structures. These structural enhancements can further improve the protective and optical characteristics of the coating. These effects have been documented in studies examining the influence of electrolyte concentration on anodic oxide films [10,11].

### 3.3. Chemical Analysis of Electrolytes as a Function of Testing Temperature

**Figure 8:**
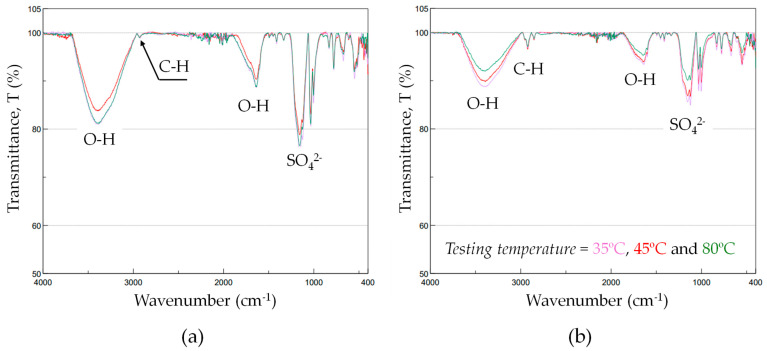
FT/IR spectra of (**a**) spectra of two aliquots used for comparison purposes of untreated solid active material (as-received condition) and (**b**) spectra obtained at different testing temperatures (35, 45 and 80 °C).

To gain a deeper understanding of the electrolyte’s behavior as a function of testing temperature, a comprehensive dynamic FTIR spectroscopy analysis was conducted. Figure 8a presents the FTIR spectrum of the untreated ion-exchange resin, while Figure 8b corresponds to the resin subjected to thermal treatment at various temperatures (35, 45, and 80 °C). The spectral range from 4000 to 400 cm^−1^ was analyzed to assess structural changes in the polymer matrix.

In the untreated resin (Figure 8a), a broad and intense band is observed around 3300–3500 cm^−1^, assigned to the O-H stretching vibrations, which originate from hydroxyl groups and adsorbed water molecules present in the resin matrix. A weaker but discernible band near 2900 cm^−1^ corresponds to C-H stretching vibrations from aliphatic -CH_2_- groups in the polymer backbone. The region between 1700 and 1500 cm^−1^ exhibits signals associated with O-H bending modes, also indicative of water presence or sulfonic acid functionalities. Additionally, a sharp and strong absorption near 1030–1050 cm^−1^ is attributed to the symmetric and asymmetric stretching modes of the sulfonate functional groups (SO_4_^2−^), characteristic of the sulfonated sites of the resin.

Upon thermal treatment (Figure 8b), significant spectral modifications are evident. The broad O-H stretching band decreases in intensity, especially as the temperature increases to 80 °C, suggesting a progressive dehydration of the resin, likely due to the loss of physically adsorbed and possibly loosely bound water. Simultaneously, the C-H stretching band near 2900 cm^−1^ becomes more prominent, likely because the diminished O-H contribution enhances the relative visibility of hydrocarbon vibrational modes.

Moreover, a noticeable shift and reduction in the O-H bending vibrations in the mid-infrared region is detected, further supporting the removal of water molecules from the resin structure. Interestingly, the SO_4_^2−^ stretching bands around 1050 cm^−1^ remain well-defined, indicating that the sulfonic acid groups are largely retained. However, subtle changes in intensity or shape at higher temperatures may suggest the onset of partial degradation or structural rearrangement of the functional groups due to thermal exposure.

Overall, these observations are consistent with thermal dehydration processes and the initial stage of polymer degradation, as thermal treatment removes water and potentially induces minor changes in the polymer matrix or its functional sites. The increasing deviation of the spectra with temperature highlights the susceptibility of the resin to structural changes and thermally induced modifications in its chemical composition, which could impact its ion-exchange performance during the anodizing processes, as shown in Figure 7c.

### 3.4. TiO_2_ Layer Thickness as a Function of the Electrolyte Operation

**Figure 9:**
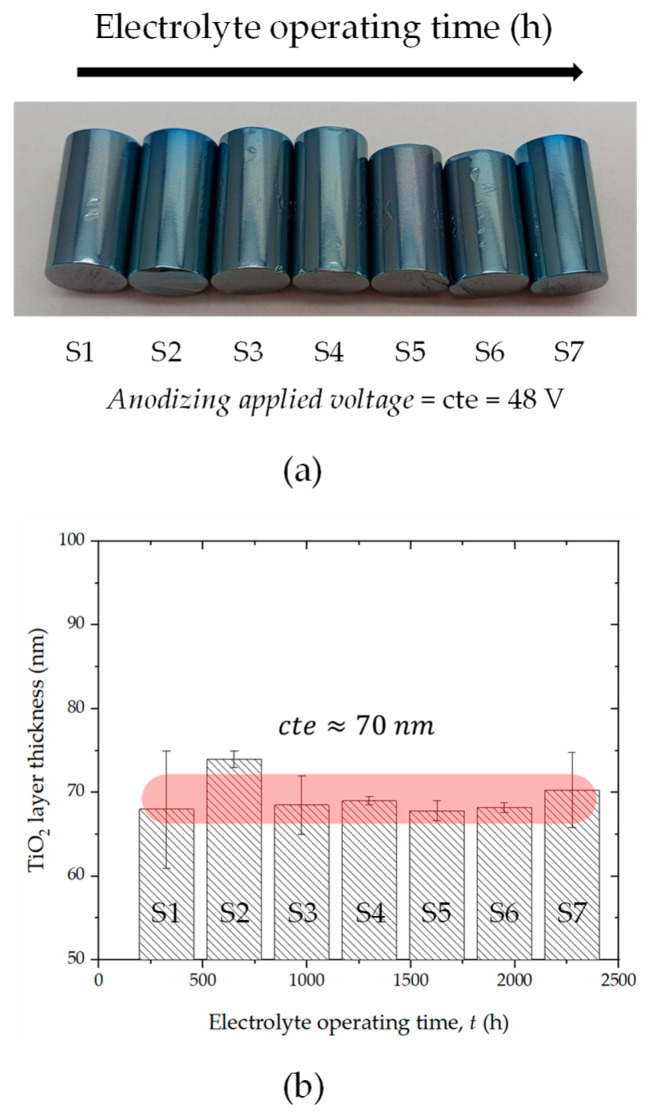
(**a**) Visual inspection of the color appearance reached when the anodizing process was held under controlled applied voltage of 48 V as a function of the electrolyte operating time and (**b**) TiO_2_ layer thickness measured using reflectometric equipment as a function of the electrolyte operating time. The semi-transparent red line highlights the uniformity of the determined TiO_2_ layer thickness.

Figure 9a presents a set of titanium cylindrical specimens anodized at 48 V for 2 min after different cycles of the anodizing process, indirectly simulating progressive resin dehydration during long-term electrolyte operation. As confirmed by previous FT/IR analysis, thermal treatment induces a reduction in the intensity of the broad O-H stretching band (see Figure 8b) and bending modes, indicating the loss of absorbed and structural water from the resin. Simultaneously, the vibration modes corresponding to the SO_4_^2−^ groups remain mostly unaffected, suggesting that the resin retains its primary ion-exchange capability even as water content diminishes.

In the context of the anodization process, resin dehydration leads to an increase in local electrolyte acidity because dehydrated resins can release protons (H^+^) more readily into the electrolyte medium. Elevated acidity levels may alter the kinetics of the oxide growth process by enhancing the dissolution of the oxide at the electrolyte/metal interface or by affecting the electrical double layer formation during anodization. However, the titanium samples in Figure 9a display a uniform interference blue coloration, characteristic of a consistent oxide thickness range of approximately 60–80 nm, indicating that the electrolyte’s capacity to support stable anodic oxide formation remains substantially preserved despite the resin’s changing hydration state.

This visual inspection is substantiated by the quantitative measurements presented in Figure 9b, where the TiO_2_ film thickness remains nearly constant at approximately 70 nm for all samples (S1 to S7), despite the increasing total electrolyte operating time (up to 2000 h) and the progressive resin aging associated with this usage time. The negligible variation in oxide thickness—as highlighted by the narrow spread within the red shaded region—suggests that the anodization process exhibits a high degree of tolerance to moderate shifts in electrolyte chemistry, specifically those induced by ion-exchange resin dehydration and potential minor degradation.

A deeper correlation with the FTIR results further clarifies this behavior. Although the resin progressively loses water—an event clearly indicated by the attenuation of the O-H absorption bands—the sulfonic acid functionalities remain structurally intact, as shown by the persistence and sharpness of the SO_4_^2−^ vibrational modes around 1050 cm^−1^ (see Figure 8b). These groups are essential for maintaining the ionic conductivity and the transport of electrolyte species during the anodization process. Thus, the electrolyte retains sufficient functional integrity to sustain anodic film growth under consistent electrochemical conditions, even if subtle acidification occurs.

Moreover, it is plausible that the enhanced acidity resulting from resin dehydration could slightly increase the field-assisted dissolution rate of the growing oxide. However, this effect may be compensated by an equally enhanced oxide formation rate at the metal/electrolyte interface, leading to a quasi-steady-state oxide thickness as reflected in the constant TiO_2_ layer measurements. This delicate balance between oxide growth and dissolution processes ensures that the overall film thickness remains stable, even as electrolyte aging progresses.

**Figure 10:**
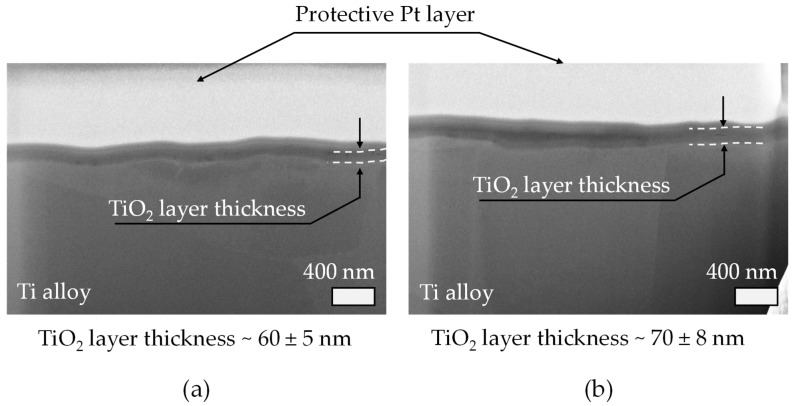
FE-SEM micrograph of the FIB cross-section conducted on samples (**a**) S1 and (**b**) S7. The white dashed line present in both FE-SEM micrographs shows the TiO_2_ layer thickness. At the bottom part of the micrograph, the TiO_2_ layer thickness is summarized as an average of 5 measurements conducted along the anodized layer generated.

Figure 10a,b display cross-sectional FIB micrographs of the anodized Ti alloy specimens corresponding to sample S1 and S7, respectively. These two samples represent the two extreme conditions regarding electrolyte usage time: S1 corresponds to the fresh electrolyte with fully hydrated resin, while S7 represents the electrolyte after prolonged operation (~2000 h) with extensively dehydrated resin, as indicated in the FTIR and anodization analysis previously discussed.

In both FIB micrographs, the presence of a homogeneous and continuous TiO_2_ oxide layer can be clearly observed, firmly adhered to the metallic Ti alloy substrate. The oxide layers exhibit comparable thicknesses, consistent with the approximate value of 70 nm measured by optical reflectometry and represented in the previous bar chart (Figure 9b). These FIB images confirm that despite electrolyte aging and resin dehydration, the anodization process yields a stable and reproducible oxide thickness, aligning with the spectroscopic FTIR evidence of preserved SO_4_^2−^ groups, which are critical for maintaining electrolyte conductivity and anodization efficiency.

A closer examination reveals subtle morphological differences between S1 and S7. In S1 (fresh resin), the TiO_2_ film appears relatively smoother and more uniform, with minimal surface roughness and a well-defined, sharp interface between the oxide and the substrate. This observation is indicative of an optimal anodization environment, where water content in the resin ensures balanced proton release and controlled oxide growth kinetics. In contrast, sample S7 (aged resin) shows a slightly rougher oxide/substrate interface and minor variations in oxide thickness across the cross-section. This is consistent with the resin dehydration detected by FTIR, where the significant reduction in O-H bands and the probable rise in electrolyte acidity may have altered the local field-assisted oxidation/dissolution balance, potentially leading to small fluctuations in oxide growth at the microscale. However, these changes do not drastically affect the average TiO_2_ thickness, which remains within the same range as S1, as shown by both the reflectometry data and FIB data presented in Figure 9b and Figure 10, respectively.

The consistency in film thickness despite resin aging reflects the robustness of the anodization process, suggesting that the electrolyte—although chemically evolving due to resin dehydration—still maintains its essential functional performance. The SO_4_^2−^ groups remain structurally intact, as revealed by the preserved SO_4_^2−^ stretching modes in the FTIR spectra, ensuring continued ion transport and current conduction during anodization.

Furthermore, the blue interference coloration observed macroscopically on the anodized Ti cylinders (Figure 9a) aligns well with the oxide thickness values visualized here by FIB, confirming the validity of optical methods for indirect oxide layer assessment. These FIB cross-sections therefore provide direct microstructural evidence supporting the earlier macroscopic and spectroscopic findings.

In summary, these FIB images validate that even under conditions of extended electrolyte operation and progressive resin dehydration, the anodic TiO_2_-layer growth remains reproducible and uniform in thickness, although minor morphological differences arise. This underscores the electrochemical system’s ability to buffer variations in electrolyte acidity induced by resin aging, as inferred from FTIR data and anodization performance metrics.

### 3.5. Corrosion Resistance

**Figure 11:**
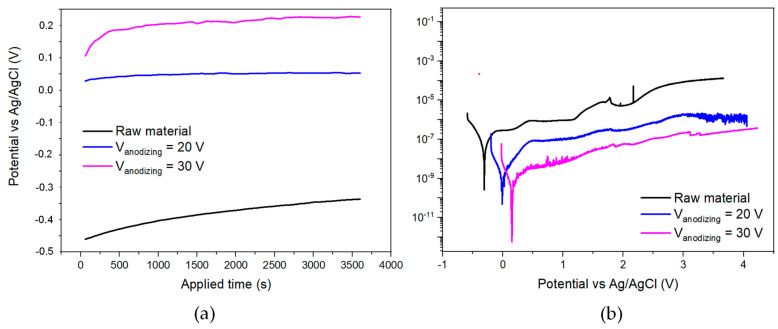
Corrosion measurements of non-anodized Ti alloy specimen and as a function of the applied voltage, 20 and 30 V for (**a**) potential vs. Ag/AgCl (y-axis) vs. the applied time (x-axis) and (**b**) current density (y-axis) as a function of the potential vs. Ag/AgCl (x-axis).

Figure 11 displays the electrical evaluation of Ti-6Al-4V alloy samples subjected to anodization treatments at different voltages (20 V and 30 V), compared to the untreated raw material, considered as a reference sample. These measurements provide valuable insight into the corrosion protection performance imparted by the anodic TiO_2_ layers whose microstructural characteristics and thickness have been previously discussed.

In Figure 11a, the evolution of the open circuit potential (OCP) versus time in Hank’s solution at 37 °C is shown. The untreated raw material exhibits a significantly lower and more negative OCP, stabilizing around −0.3 V vs. Ag/AgCl, indicative of its inherent susceptibility to corrosion in the used medium. In contrast, both anodized samples exhibit a marked positive shift in OCP values, with stabilization at approximately 0.0 V for 20 V anodized samples and +0.15 V for 30 V anodized samples. This positive displacement reflects the formation of a stable and protective TiO_2_ barrier layer, which effectively reduces the thermodynamic driving force for corrosion initiation. The higher OCP for the 30 V anodized sample correlates well with its thicker oxide layer, previously measured by reflectometry and FIB as being close to 70 nm.

Figure 11b shows potentiodynamic polarization curves recorded in the same electrolyte. The raw material displays a higher corrosion current density, and a noisy curve indicative of active corrosion processes, including pitting initiation. Conversely, the anodized samples exhibit two orders of magnitude lower current densities, reaching values below 10^−9^ A/cm^2^ for the 20 V sample and even down to 10^−11^ A/cm^2^ for the 30 V sample in the passive region. The significantly reduced anodic current densities in the passivation range are direct evidence of the superior corrosion resistance conferred by the anodic oxide layers.

These electrochemical improvements strongly correlate with the thickness and integrity of the TiO_2_ layers revealed by the FIB cross-sectional analysis. As demonstrated in previous studies [11], the anodic films formed under both 20 and 30 V conditions are continuous and compact, with an average thickness close to 70 nm for the 30 V sample. The thicker oxide layer offers enhanced physical and ionic barrier properties, impeding the ingress of ions and thereby lowering the corrosion current. Additionally, the FT/IR spectra confirm that the electrolyte maintains sufficient functional group integrity (SO_4_^2−^) to support consistent oxide growth even during extended operation, explaining the reproducibility of the oxide’s protective properties.

The stabilization of corrosion performance is also consistent with the minimal variation in oxide thickness between the different electrolyte aging stages, as reflected in the previous reflectometry data (constant ~70 nm). This suggests that the anodization process is robust enough to tolerate moderate electrolyte degradation without significant loss of corrosion resistance. However, the best performance (lowest current density, highest OCP) is clearly achieved with the sample anodized at the higher voltage (30 V), where a thicker and more protective TiO_2_ film is formed.

## 4. Conclusions

The effect of temperature through the dry anodizing process on the Ti6Al4V alloy was investigated. The following conclusions may be drawn:

(1) Controlled thermal exposure of the solid polymer electrolyte at temperatures above 40 °C induces notable morphological and chemical transformations, primarily driven by dehydration mechanisms.
(2) A saturation threshold is reached at ~40–45 °C, beyond which further thermal exposure does not significantly increase particle degradation, indicating a thermally equilibrated state.
(3) Joule heating causes deformation of the polymer particles, leading to loss of spherical geometry and the formation of surface microcracks due to thermal fatigue.
(4) The particle size distribution evolves from bimodal to trimodal after thermal aging at 45 °C, accompanied by a reduction in both fine and coarse particle diameters by approximately 39.5 and 22.8%, respectively. This is attributed to dehydration and possible particle fragmentation.
(5) FTIR analysis reveals a progressive reduction in O-H stretching and bending vibrations with increasing temperature, confirming dehydration. However, sulfonate functional groups remain largely intact, preserving the ion-exchange functionality of the resin.
(6) The anodization process using ion-exchange resin-conditioned electrolytes exhibits remarkable resilience to the effects of resin aging and dehydration, as detected by FT/IR, ensuring stable and reproducible oxide layer formation throughout the electrolyte’s operational life.
(7) Electrolyte dehydration leads to an increase in local acidity within the particles, which enhances the anodizing process by facilitating higher current densities and improving TiO_2_ layer formation.
(8) Despite thermal aging and resin dehydration, the TiO_2_ layer thickness remains stable (~70 nm) even after prolonged electrolyte usage (up to 2000 h), demonstrating the robustness and reliability of the process.
(9) The electrolyte maintains sufficient chemical and structural integrity to support consistent electropolishing and anodizing performance despite the microstructural and compositional changes induced by Joule heating and thermal aging.
(10) The observed balance between oxide growth and dissolution processes ensures the formation of uniform, high-quality TiO_2_ layers over extended operational periods.
(11) Samples anodized at higher voltages (30 V) exhibit superior electrochemical stability, as indicated by higher open circuit potentials and lower corrosion current densities. This improvement directly correlates with the increased oxide thickness (~70 nm), which serves as an effective barrier against the used medium simulating in vivo conditions.
(12) Reflectometry and FIB analyses confirm that the oxide layer thickness remains stable across various electrolyte aging stages, explaining the consistent corrosion resistance behavior even after prolonged electrolyte use.

## Figures and Tables

**Table 1:** Summary of the particle size distribution for the untreated and thermally aged particles at 45 °C, determined using a uniform bin size of 25 μm, based on a dataset of 1200 measured particles and analyzed using the statistical methodology proposed by the Ulm and Constantinides method [15,16,17]. The parameter f_i_ represents the relative fraction (in %) of each particle type within the total particle content of the solid polymer electrolyte medium.

**Untreated**			
	**Fine**	**Medium**	**Coarse**
Ø_i_ (μm)	960 ± 540	-	1481 ± 481
f_i_ (%)	80.4	-	20.6
χ_i_^2^ (-)	1.04 × 10^−6^	-	5.58 × 10^−7^
R_i_^2^ (-)	0.99999	-	0.99998
**Thermally Aged Particles at 45 °C**			
	**Fine**	**Medium**	**Coarse**
Ø_i_ (μm)	581 ± 100	765 ± 300	1143 ± 75
f_i_ (%)	23.8	45.4	30.8
χ_i_^2^ (-)	7.50 × 10^−6^	4.36 ×·10^−6^	9.66 × 10^−6^
R_i_^2^ (-)	0.99910	0.99995	0.99988

## Data Availability

The original contributions presented in this study are included in the article. Further inquiries can be directed to the corresponding author.

## Abbreviations

The following abbreviations are used in this manuscript:

AC	Alternating current
CsI	Cesium iodide
C-H	Hydrocarbon group
-CH_2_-	Aliphatic group
DSC	Differential scanning calorimetry
FIB/FESEM	Focused ion beam/field emission scanning electron microscope
FT/IR	Fourier transform infrared spectroscopy
Ga^+^	Gallium ion
H-H_2_SO_4_	High-concentration sulfuric acid electrolyte
H_2_SO_4_	Sulfuric acid
O-H	Hydroxyl groups
SO_4_^2−^	Sulfate functional group
TiO_2_	Titanium dioxide
T^+^	Positive pulse time
T^−^	Negative pulse time
Tp	Pause time
TGA	Thermogravimetric analyzer
UV	Ultraviolet
Pt	Platinum
3D	Three-dimensional

## References

[1] Zhao Q., Sun Q., Xin S., Chen Y., Wu C., Wang H., Xu J., Wan M., Zhen W., Zhao Y. (2022). High-strength titanium alloys for aerospace engineering applications: A review on melting-forging process. Mat. Sci. Eng. A.

[2] Rack H., Qazi J.I. (2006). Titanium alloys for biomedical applications. Mat. Sci. Eng. C..

[3] Mahyar Khorasani A., Goldberg M., Coeven E.H., Littlefair G. (2015). Titanium in Biomedical Applications—Properties and Fabrication: A review. J. Biomater. Tiss. Eng..

[4] Saurabh A., Madhu Meghana C., Kumar Singh P., Chandra Verma P. (2022). Titanium-based materials: Synthesis, properties, and applications. Mat. Today Proc..

[5] Liu S., Shin C. (2019). Additive manufacturing of Ti6Al4V alloy: A review. Mater. Des..

[6] Cui C., Hu B., Zhao L., Liu S. (2011). Titanium alloy production technology, market prospects and industry development. Mater. Des..

[7] Huang R., Riddle M., Graziano D., Warren J., Das S., Nimbalkar S., Cresko J., Masanet E. (2016). Energy and emissions saving potential of additive manufacturing: The case of lightweight aircraft components. J. Clean. Prod..

[8] Vermessea E., Mabrua C., Aruraultb L. (2013). Surface integrity after pickling and anodization of Ti-6Al-4V titanium alloy. Appl. Surf. Sci..

[9] Zhecheva A., Sha W., Malinov S., Long A. (2005). Enhancing the microstructure and properties of titanium alloys through nitriding and other surface engineering methods. Surf. Coat. Technol..

[10] Cigada A., Carbini M., Pepeferri P. (1992). Increasing the corrosion resistance of the Ti6Al4V alloy by high thickness anodic oxidation. J. Mater. Sci. Mater. Med..

[11] Valencia-Cadena A., Garcia-Blanco M.B., Reschenhofer B., Barreneche C., Skerbis P., Leitl P.A., Santamaria P., Roa J.J. (2025). In-depth study of the dry anodizing process on Ti6Al4V alloys: Effect of the acid content and electrical parameters. Surf. Coat. Technol..

[12] Crittenden J.C., Truseel R.R., Hand D.W., Howe K.J., Tchobanoglous G. (2012). MWH’s Water Treatment: Principles and Design.

[13] Fathi M.B., Rezai B., Alamdari E.K., Alorro R.D. (2017). Mechanisms and equilibrium modeling of Re and Mo adsorption on a gel type strong base anion resin. Rus. J. Appl. Chem..

[14] Charrière R., Cridling Q., Maillet M., Pedeferri M.P., Delafosse D. (2020). Determination of oxidized metals’ oxide layer thickness from local extrema of reflectance spectra: Theoretical basis and application to anodized titanium. Meas. Sci. Technol..

[15] Ulm F.J., Vandamme M., Bobko C., Alberto Ortega J., Tai K., Ortiz C. (2007). Statistical indentation techniques for hydrated nanocomposites: Concrete, bone, and shale. J. Am. Ceram. Soc..

[16] Constantinides G., Ulm F.J., Van Vliet K. (2003). On the use of nanoindentation for cementitious materials. Mater. Struct..

[17] Constantinides G., Ravi Ghandran K.S., Ulm F.J., Van Vliet K.J. (2006). Grid indentation analysis of composite microstructure and mechanics: Principles and validation. Mater. Sci. Eng. A.

